# Sexually dimorphic swallows have higher extinction risk

**DOI:** 10.1002/ece3.3723

**Published:** 2017-12-12

**Authors:** Masaru Hasegawa, Emi Arai

**Affiliations:** ^1^ Department of Evolutionary Studies of Biosystems Sokendai (The Graduate University for Advanced Studies) Miura‐gun Kanagawa Japan

**Keywords:** extinction, sexual plumage dimorphism, swallows, threatened species

## Abstract

The effect of sexual selection on extinction risk remains unclear. In theory, sexual selection can lead to both increase and decrease extinction probability depending on the ecology of the study system. Thus, combining different groups might obscure patterns that can be found in groups that share similar ecological features. Using phylogenetic comparative analysis, we studied sexual plumage dimorphism in relation to the perceived risk of extinction in hirundines (subfamily: Hirundininae), in which all species are socially monogamous aerial foragers. Among the 72 species studied, five species are facing a perceived threat of extinction. Species with sexually dimorphic plumage had a higher risk of extinction than did species with sexually monomorphic plumage. Likewise, when focusing solely on tail ornamentation, species that exhibit a sexual dimorphism in tail length had a higher risk of extinction than did other species. In Hirundininae, which are affected a great deal by severe weather, sexual selection and the resultant sexual dimorphism would increase extinction risk.

## 1 INTRODUCTION

The effect of sexual selection on extinction risk attracts the attention of modern evolutionary biologists, because the direction in which sexual selection affects extinction risk is not straightforward at least in theory (e.g., Candolin & Heuschele, 2008; Kokko & Brooks, 2003; Martínez‐Ruiz & Knell, 2017). For example, Martínez‐Ruiz and Knell (2017) recently showed that whether sexual selection exerts a positive or negative effect on extinction risk depends on several factors, such as the condition dependence of sexually selected traits, fecundity, and nature of environmental variability (also see Kokko & Brooks, 2003 for high extinction risk when sexual selection results in fecundity costs to females). This indicates that sexual selection did not have the same effect in all study systems. Rather, the specific details of the ecology of the study system will determine whether sexual selection leads to positive or negative effect on extinction risk (Martínez‐Ruiz & Knell, 2017).

Using a phylogenetic comparative analysis, Morrow and Pitcher (2003) found no detectable relationship between sexual plumage dimorphism and extinction risk (i.e., an index of the perceived risk of extinction based on the International Union for the Conservation of Nature (IUCN) Red List; Fisher & Owens, 2004) in birds. Because they found a significant positive association between testis size and extinction risk, they concluded that sexual plumage dimorphism, and thus sexual selection of plumage characteristics, should be less important. Still, as noted above, several factors affect the impact of sexual selection on extinction risk, and thus combining different groups might obscure patterns that can be found in groups that share similar ecological features. This is already pointed out in the analysis of the fate of birds introduced to islands, in which the relationship between sexual selection and introduction success is taxon‐specific (e.g., McLain, Moulton, & Redfern, 1995; Moulton, Mclain, & Moulton, 2009; Sorci, Møller, & Clobert, 1998). The relationship between sexual selection and extinction risk should be studied using a clade with similar ecological features (also see Bro‐Jørgensen, 2014; and citations therein for the taxon specificity of predictors of extinction risk in mammals).

Here, we investigated the relationship between sexual plumage dimorphism and extinction risk in hirundines (subfamily: Hirundininae). In the Hirundininae, all species are socially monogamous with biparental care (Turner & Rose, 1994). Moreover, they are highly specialized to aerial foraging and thus may experience different selection pressures compared to the rest of passerines. In fact, Huber, Turbek, Bostwick, and Safran (2017) recently found a reversal of the correlation between migration and wing shape (pointedness, here) found in other avian taxa, indicating the importance of taxon‐specific analysis in these aerial insectivores. Although sexual selection on socially monogamous species is classically thought to be weak, this is not always the case (Andersson, 1994). As is shown in the barn swallow, *Hirundo rustica*, a well‐known model species of sexual selection, sexually selected ornamentation can evolve and be maintained by several sexual selection mechanisms, including the Darwin–Fisher mechanism (i.e., benefits of early breeding by attractive males), extrapair paternity, and differential parental investment (i.e., females paired with attractive males had higher reproductive investment: reviewed in Møller, 1994, 2003; Turner, 2006). Intense sexual selection associated with these mechanisms may shape the evolution of sexual dimorphism (Møller, 2003).

Using a phylogenetic comparative analysis, we first studied the relationship between sexual plumage dimorphism (in all plumage‐based characteristics) and extinction risk. Then, because long tails have been acquired and lost repeatedly in this clade (Johnson, Mitchell, & Brown, 2016), and because long tails are well‐known sexually selected traits at least in the barn swallow *H. rustica* (see above), we also determined whether sexual tail dimorphism explained extinction risk. Hirundines are affected great deal by environmental changes, because they require adequate weather to feed (e.g., heavy rain, drought, and cyclone lead to population decline, population crash, and large‐scale mortality; reviewed in Turner & Rose, 1994). Such a drastic environmental effect would be particularly costly in highly ornamented species (e.g., longer‐tailed swallows exhibit reduced aerodynamic ability and thus have survival and fecundity costs; Møller, 1994; Hasegawa & Arai, 2017). For these reasons, we predicted that sexually dimorphic hirundines have a higher extinction risk than do monomorphic hirundines.

## 2 MATERIALS AND METHODS

### 2.1 Data collection

As in previous studies (Hasegawa & Arai, 2017; Hasegawa, Arai, & Kutsukake, 2016), morphological information (i.e., wing length, and sexual dimorphism/monomorphism in wing and tail length: note that male's wing length was the only measurement used, while sexual dimorphism/monomorphism is binary variable) and migratory habits (migrants and others) were obtained from Turner and Rose (1994). Detailed information is provided in previous studies (Hasegawa & Arai, 2017; Hasegawa et al., 2016). For sexual plumage dimorphism, we used plates in del Hoyo and Collar (2016), because Turner and Rose (1994) lack some illustrations of males and females, even when they markedly differed (e.g., *Hirundo smithii*). Species were classified as sexually dimorphic when males and females had distinctive plumage, that is, when both male and female are depicted in the plates in del Hoyo and Collar (2016). In the plate of del Hoyo and Collar (2016), a single bird in breeding plumage is depicted for species with little or no sexual dimorphism while both male and female are depicted in breeding plumage for sexually dimorphic species (i.e., we followed this criteria: see del Hoyo & Collar, 2014 for the explanation). An alternative approach using the degree of sexual dimorphism as a continuous variable, for example, log(male tail length) −log(female tail length), was not adopted in the current case, because the accurate estimate is difficult to obtain from species with scarce information, and because sex‐specific information cannot be obtained from some “monomorphic” species even when they show some degree of sexual dimorphism in measurements (note that, when multiple traits such as tail fork depth and plumage coloration are involved, degree of sexual dimorphism is difficult to compare even in well‐known species: For example, whether *H. smithii* has a higher degree of sexual dimorphism than *Progne subis*). We also collected information from the IUCN red list using del Hoyo and Collar (2016). We regarded “threatened species” (i.e., critically endangered, endangered, and vulnerable) as species facing a perceived threat of extinction, as in previous studies (Morrow & Pitcher, 2003; reviewed in Fisher & Owens, 2004). Other species (i.e., least concerns) were deemed at lower risk (note that near threatened is absent in the current data set). In total, we obtained data from all 72 species listed in Turner and Rose (1994). The data set for this study is given in Table S1.

### 2.2 Phylogenetic comparative analysis

We conducted phylogenetic logistic regression analyses using the function phyloglm in the R package “phylolm” (Ho & Ané, 2014). To account for phylogenetic uncertainty, we fitted the models to each tree and applied multimodel inference using 1,000 alternative trees of swallows from birdtree.org (Garamszegi & Mundry, 2014; Hasegawa & Arai, 2017). We derived model‐averaged mean coefficients, *SE*s, and 95% confidence intervals (CIs) via model averaging. We also presented the α value, which is associated with phylogenetic signals with larger values indicating greater rates of transitions (Ives & Garland, 2010). To visually check replicated co‐distribution (Maddison & FitzJohn, 2016), we also presented an example of ancestral character reconstruction using the functions “ace” in the R package “ape” and “plotTree” in the R package “phytools” (Revell, 2012). Although there are only a few vulnerable species (*N *=* *5), within‐clade pseudoreplication should be negligible in the current case, because both sexual plumage dimorphism and vulnerable species appear scattered in the Hirundininae (i.e., not clustered in a specific clade). In fact, when we applied a threshold model to the subset of 1,000 alternative trees (function “threshBayes” in the R package “phytools”), which is immune to within‐clade pseudoreplication, we obtained qualitatively similar results (i.e., a significant effect of sexual plumage dimorphism, although sexual tail dimorphism can be marginal in some cases; see Figure S1 for some examples). When we investigated the variance inflation factor (VIF) values using function “vif” in the R package “car” (Fox & Weisberg 2011), max VIF was 2.55, and thus, there was no clear indication of a problem (VIF >10 is problematic; Mundry, 2014).

## 3 RESULTS

**Figure 1:**
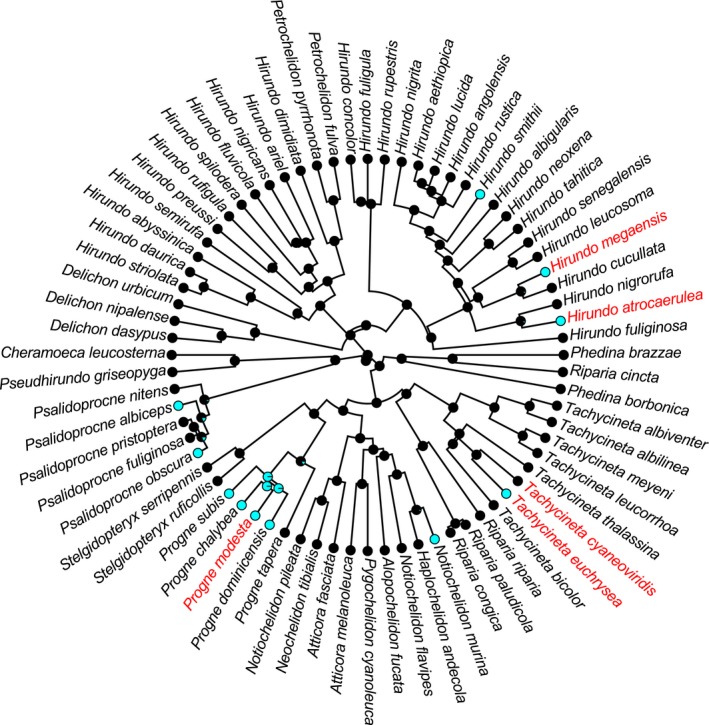
Example of ancestral character reconstruction of sexual plumage dimorphism. Cyan (pale gray in print) and black circles at tips indicate sexually dimorphic and monomorphic species, respectively. Likewise, the proportions of cyan (pale gray in print) and black in nodes indicate the probability of sexually dimorphic and monomorphic states. Vulnerable species are indicated in red (gray in print)

Among the 72 species studied, five species are facing a perceived threat of extinction, and four of them exhibit obvious sexual plumage dimorphism (Figure 1). We found a significant association between sexual plumage dimorphism and the perceived risk of extinction (Table 1a): Species with sexual plumage dimorphism had a higher extinction risk than did other species (also see Figure 1). The odds ratio of the relationship was 17.99, indicating that sexually dimorphic species had ca. 18 times higher probability to be threated than monomorphic species. Although we included two potential confounding variables, log(wing length) and migratory habits (i.e., migrants or not), these variables were not significant (Table 1a).

**Table 1:** Multivariable phylogenetic logistic regression model predicting the probability of vulnerability to extinction in relation to (a) sexual plumage dichromatism and (b) sexual size and tail dimorphism (both: *N *=* *72)

	Coefficient ± *SE*	95% CI
(a) Overall plumage		
Sexual plumage dimorphism	**2.89 ± 1.02**	**0.89–4.89**
log (wing length)	1.75 ± 4.02	−6.12–9.62
Migratory habits (migrants = 1)	−1.16 ± 1.08	−3.28–0.96
		Model‐averaged alpha value = 0.16
(b) Wing and tail dimorphism		
Sexual dimorphism in wing length	1.94 ± 3.26	−4.45–8.34
Sexual dimorphism in tail length	**2.49 ± 1.24**	**0.06–4.92**
log (wing length)	−3.19 ± 4.35	−11.72–5.34
Migratory habits (migrants = 1)	−1.96 ± 3.18	−8.20–4.28
		Model‐averaged alpha value = 0.20

Model‐averaged coefficients, *SE*, and 95% confidence intervals (CI) are shown. Significant results (i.e., 95% CI does not contain zero) are indicated in bold.

We further studied whether sexual dimorphism in tail length can explain extinction risk. For this purpose, we included sexual wing dimorphism as a covariate to account for sexual size dimorphism. In this analysis, we found that species with sexual tail dimorphism, but not sexual wing dimorphism, had a higher extinction risk (Table 1b). The odds ratio of the relationship was 12.06, indicating that sexually dimorphic species had ca. 12 times higher probability to be threated than monomorphic species. Again, log(wing length) and migratory habits were not significant (Table 1b).

## 4 DISCUSSION

As predicted, we found that species with sexually dimorphic plumage have a higher extinction risk in the Hirundininae, in part due to the sexually dimorphic tail length. We controlled for phylogenetic inertia, body size, and migratory habits; thus, these variables might not confound the observed pattern. This observation contrasts with Morrow and Pitcher (2003), in which relationship between sexual dimorphism and extinction risk was not detected across birds. The discrepancy between the two studies can be explained if combining different groups might obscure patterns that can be found in groups that share similar ecological features (i.e., hirundines, here; Martínez‐Ruiz & Knell, 2017).

A simple theoretical model suggests that a catastrophe may lead to extinction of species with sexually selected traits, while nonsexually selected species persist (Kokko & Brooks, 2003). Rare, severe weather leads to population decline, population crash, and large‐scale mortality in hirundines that are aerial insectivores (Turner & Rose, 1994), and such catastrophic events might drive sexually dimorphic species to face a higher perceived risk of extinction due to the cost of ornamentation (e.g., reduced aerodynamic ability of long‐tailed swallows; also see Møller, 2000 for a similar argument). Although the burden of bearing ornamentation is not added to that of environmental stress when species plastically reduce ornamentation expression in response to environmental stress (via condition dependence; Martínez‐Ruiz & Knell, 2017), this is not the case in hirundines. They do experience sudden environmental changes (see above), and their plumage ornamentation has limited plasticity after the molting period.

Also, intra‐ and interlocus sexual conflict might matter (Kokko & Brooks, 2003): If sexually selected traits, which are beneficial in males result in fecundity costs to females, it may lead to extinction. Ornamentation that trades off with reproductive investment (e.g., provisioning of nestlings; Møller, 1994) should constrain the evolution and maintenance of female ornamentation. This intralocus sexual conflict can be partially resolved by the evolution of sexual dimorphism (Chenoweth, Doughty, & Kokko, 2006; Fitzpatrick, Berglund, & Rosenqvist, 1995). However, in hirundines, which are socially monogamous with biparental care (Turner & Rose, 1994), females paired with highly ornamented males should have additional costs due to the low reproductive investment of mates (i.e., interlocus sexual conflict). Such costs would be particularly high during severe weather (see above). It remains to be clarified whether similar pattern can be found in other taxa with similar ecological features (e.g., swifts). Lastly, sexually dimorphic clades might have more speciation events, and the resultant young species might have a higher perceived risk of extinction. This explanation is unlikely in the current case, as the branch length of species with and without extinction risk is not so different (Figure 1).

In the current study, we found an association between sexual plumage dimorphism and extinction risk in the Hirundininae, which contrasts with no detectable relationship between sexual dimorphism and extinction risk when different groups of birds are combined (Morrow & Pitcher, 2003). Shared ecological features of the Hirundininae, such as aerial foraging and social monogamy, might explain the relationship. Nonetheless, our data set could not clarify the relative importance of these and other factors due to the limited information available (e.g., fecundity, inbreeding depression, Allee effects, and so on), which remains to be clarified using other taxa.

## AUTHOR CONTRIBUTION

MH and EA collected the data and wrote the manuscript together.

## References

[ece33723-bib-0001] Andersson, M. (1994). Sexual selection. Princeton, NJ: Princeton University Press.

[ece33723-bib-0002] Bro‐Jørgensen, J. (2014). Will their armaments be their downfall? Large horn size increases extinction risk in bovids. Animal Conservation, 17, 80–87. https://doi.org/10.1111/acv.12062

[ece33723-bib-0003] Candolin, U. , & Heuschele, J. (2008). Is sexual selection beneficial during adaptation to environmental change?. Trends in Ecology & Evolution, 23, 446–452. https://doi.org/10.1016/j.tree.2008.04.008 18582989 10.1016/j.tree.2008.04.008

[ece33723-bib-0004] Chenoweth, S. F. , Doughty, P. , & Kokko, H. (2006). Can non‐directional male mating preferences facilitate honest female ornamentation? Ecology Letters, 9, 179–184. https://doi.org/10.1111/j.1461-0248.2005.00867.x 16958883 10.1111/j.1461-0248.2005.00867.x

[ece33723-bib-0005] del Hoyo, J. , & Collar, N. J. (2014). HBW and BirdLife International Illustrated Checklist of the Birds of the World. Vol. 1: Non‐Passerines. Barcelona, Spain: Lynx Edicions.

[ece33723-bib-0006] del Hoyo, J. , & Collar, N. J. (2016). HBW and BirdLife International Illustrated Checklist of the Birds of the World. Vol. 2: Passerines. Barcelona, Spain: Lynx Edicions.

[ece33723-bib-0007] Fisher, D. O. , & Owens, I. P. F. (2004). The comparative method in conservation biology. Trends in Ecology & Evolution, 19, 391–398. https://doi.org/10.1016/j.tree.2004.05.004 16701291 10.1016/j.tree.2004.05.004

[ece33723-bib-0008] Fitzpatrick, S. , Berglund, A. , & Rosenqvist, G. (1995). Ornaments or offspring: Costs to reproductive success restrict sexual selection processes. Biological Journal of the Linnean Society, 55, 251–260. https://doi.org/10.1111/j.1095-8312.1995.tb01063.x

[ece33723-bib-0600] Fox, J. , & Weisberg, S. (2011). An R companion to applied regression 2nd edn. Sage Publications: Thousand Oaks.

[ece33723-bib-0009] Garamszegi, L. Z. , & Mundry, R. (2014). Multimodel‐inference in comparative analyses In Garamszegi L. Z. (Ed.), Modern phylogenetic comparative methods and their application in evolutionary biology: Concepts and practice (pp. 305–331). New York, NY: Springer.

[ece33723-bib-0010] Hasegawa, M. , & Arai, E. (2017). Egg size decreases with increasing female fork tail in family Hirundinidae. Evolutionary Ecology, 31, 559–569. https://doi.org/10.1007/s10682-017-9895-2

[ece33723-bib-0011] Hasegawa, M. , Arai, E. , & Kutsukake, N. (2016). Evolution of tail fork depth in genus *Hirundo* . Ecology and Evolution, 6, 851–858. https://doi.org/10.1002/ece3.1949 26865972 10.1002/ece3.1949 PMC4739571

[ece33723-bib-0012] Ho, L. S. T. , & Ané, C. (2014). A linear‐time algorithm for Gaussian and non‐Gaussian trait evolution models. Systematic Biology, 63, 397–408. 24500037 10.1093/sysbio/syu005

[ece33723-bib-0013] Huber, G. H. , Turbek, S. P. , Bostwick, K. S. , & Safran, R. J. (2017). Comparative analysis reveals migratory Swallows (Hirundinidae) have less pointed wings than residents. Biological Journal of the Linnean Society, 120, 228–235. https://doi.org/10.1111/bij.12875

[ece33723-bib-0014] Ives, A. R. , & Garland, Jr, T. G. (2010). Phylogenetic logistic regression for binary dependent variables. Systematic Biology, 59, 9–26. https://doi.org/10.1093/sysbio/syp074 20525617 10.1093/sysbio/syp074

[ece33723-bib-0015] Johnson, A. E. , Mitchell, J. S. , & Brown, M. B. (2016). Convergent evolution in social Swallows (Aves: Hirundinidae). Ecology and Evolution, 7, 550–560. 28116052 10.1002/ece3.2641 PMC5243784

[ece33723-bib-0016] Kokko, H. , & Brooks, R. (2003). Sexy to die for? Sexual selection and the risk of extinction. Annales Zoologici Fennici, 40, 207–219.

[ece33723-bib-0017] Maddison, W. P. , & FitzJohn, R. G. (2016). The unsolved challenge to phylogenetic correlation tests for categorical characters. Systematic Biology, 64, 127–136. 10.1093/sysbio/syu070 25209222

[ece33723-bib-0018] Martínez‐Ruiz, C. , & Knell, R. J. (2017). Sexual selection can both increase and decrease extinction probability: Reconciling demographic and evolutionary factors. Journal of Animal Ecology, 86, 117–127. https://doi.org/10.1111/1365-2656.12601 27861841 10.1111/1365-2656.12601

[ece33723-bib-0019] McLain, D. K. , Moulton, M. P. , & Redfern, T. P. (1995). Sexual selection and the risk of extinction of introduced birds on oceanic islands. Oikos, 74, 271–281.

[ece33723-bib-0020] Møller, A. P. (1994). Sexual selection and the barn swallow. Oxford, UK: Oxford University Press.

[ece33723-bib-0021] Møller, A. P. (2000). Sexual selection and conservation In Gosling L. M. & Sutherland W. J. (Eds.), Behaviour and conservation (conservation biology) (pp. 161–171). Cambridge, UK: Cambridge University Press.

[ece33723-bib-0022] Møller, A. P. (2003). The evolution of monogamy: Mating relationships, parental care and sexual selection In Reichard U. H. & Boesch C. (Eds.), Monogamy: Mating strategies and partnerships in birds, humans and other mammals (pp. 29–41). Cambridge, UK: Cambridge University Press https://doi.org/10.1017/CBO9781139087247

[ece33723-bib-0023] Morrow, E. H. , & Pitcher, T. E. (2003). Sexual selection and the risk of extinction in birds. Proceedings of the Royal Society of London, Series B: Biological Sciences, 270, 1793–1799. https://doi.org/10.1098/rspb.2003.2441 12964981 10.1098/rspb.2003.2441 PMC1691441

[ece33723-bib-0024] Moulton, M. P. , Mclain, D. K. , & Moulton, L. E. (2009). Sexual selection and the fate of introduced pigeons and doves (Aves: Columbidae). Evolutionary Ecology Research, 11, 889–904.

[ece33723-bib-0025] Mundry, R. (2014). Statistical issues and assumptions of phylogenetic generalized least squares In Garamszegi L. Z. (Ed.), Modern phylogenetic comparative methods and their application in evolutionary biology: Concepts and practice (pp. 131–153). New York, NY: Springer.

[ece33723-bib-0027] Revell, L. J. (2012). phytools: An R package for phylogenetic comparative biology (and other things). Methods in Ecology and Evolution, 3, 217–223. https://doi.org/10.1111/j.2041-210X.2011.00169.x

[ece33723-bib-0030] Sorci, G. , Møller, A. P. , & Clobert, J. (1998). Plumage dichromatism of birds predicts introduction success in New Zealand. Journal of Animal Ecology, 67, 263–269. https://doi.org/10.1046/j.1365-2656.1998.00199.x

[ece33723-bib-0320] Turner, A. K. (2006). The barn swallow. T & A D Poyser: London.

[ece33723-bib-0032] Turner, A. K. , & Rose, C. (1994). A handbook to the swallows and martins of the world. London, UK: Christopher Helm.

