# Supplementary material for: Sexually dimorphic swallows have higher extinction risk
Source: Ecol Evol. 2017 Dec 12;8(2):992–6. doi: 10.1002/ece3.3723 (PMC5773298; doi:10.1002/ece3.3723)
Supplement: Supplementary file 1 [file ECE3-8-992-s001.docx]

Table S1 Data set of the current study of the family Hirundininae.

| Species | Male wing length | Migratory habit | Risk of extinction | Sexual dimorphism | | |
| --- | --- | --- | --- | --- | --- | --- |
|  |  |  |  | Overall plumage | Wing length | Tail length |
| *Neochelidon tibialis* | 85 | 0 | 0 | 0 | 0 | 0 |
| *Alopochelidon fucata* | 100 | 1 | 0 | 0 | 0 | 0 |
| *Stelgidopteryx ruficollis* | 109 | 1 | 0 | 0 | 1 | 1 |
| *Stelgidopteryx serripennis* | 110 | 1 | 0 | 0 | 1 | 1 |
| *Tachycineta bicolor* | 119 | 1 | 0 | 0 | 1 | 0 |
| *Tachycineta albilinea* | 97 | 0 | 0 | 0 | 1 | 0 |
| *Tachycineta albiventer* | 104 | 1 | 0 | 0 | 0 | 0 |
| *Tachycineta thalassina* | 122 | 1 | 0 | 0 | 1 | 0 |
| *Tachycineta leucorrhoa* | 115 | 1 | 0 | 0 | 0 | 0 |
| *Tachycineta meyeni* | 110 | 1 | 0 | 0 | 0 | 0 |
| *Tachycineta cyaneoviridis* | 115 | 0 | 1 | 0 | 1 | 1 |
| *Tachycineta euchrysea* | 106 | 0 | 1 | 1 | 0 | 0 |
| *Notiochelidon murina* | 111 | 0 | 0 | 1 | 0 | 0 |
| *Notiochelidon flavipes* | 90 | 0 | 0 | 0 | 0 | 0 |
| *Notiochelidon pileata* | 95 | 0 | 0 | 0 | 0 | 0 |
| *Pygochelidon cyanoleuca* | 94 | 1 | 0 | 0 | 0 | 0 |
| *Atticora fasciata* | 101 | 0 | 0 | 0 | 0 | 0 |
| *Atticora melanoleuca* | 93 | 0 | 0 | 0 | 0 | 0 |
| *Progne tapera* | 130 | 1 | 0 | 0 | 1 | 0 |
| *Progne subis* | 145 | 1 | 0 | 1 | 1 | 1 |
| *Progne chalybea* | 131 | 1 | 0 | 1 | 1 | 1 |
| *Progne dominicensis* | 143 | 1 | 0 | 1 | 1 | 1 |
| *Progne modesta* | 125 | 1 | 1 | 1 | 1 | 1 |
| *Riparia paludicola* | 104 | 0 | 0 | 0 | 0 | 0 |
| *Riparia riparia* | 107 | 1 | 0 | 0 | 0 | 0 |
| *Riparia cincta* | 130 | 1 | 0 | 0 | 0 | 0 |
| *Riparia congica* | 92 | 0 | 0 | 0 | 0 | 0 |
| *Psalidoprocne fuliginosa* | 104 | 0 | 0 | 0 | 0 | 0 |
| *Psalidoprocne albiceps* | 102 | 1 | 0 | 1 | 0 | 1 |
| *Psalidoprocne pristoptera* | 106 | 1 | 0 | 0 | 0 | 1 |
| *Psalidoprocne obscura* | 96 | 0 | 0 | 1 | 0 | 1 |
| *Psalidoprocne nitens* | 93 | 0 | 0 | 0 | 0 | 0 |
| *Cheramoeca leucosterna* | 102 | 0 | 0 | 0 | 0 | 0 |
| *Pseudhirundo griseopyga* | 97 | 0 | 0 | 0 | 0 | 0 |
| *Phedina borbonica* | 116 | 0 | 0 | 0 | 0 | 0 |
| *Phedina brazzae* | 100 | 0 | 0 | 0 | 0 | 0 |
| *Hirundo rupestris* | 130 | 1 | 0 | 0 | 0 | 0 |
| *Hirundo fuligula* | 129 | 0 | 0 | 0 | 0 | 0 |
| *Hirundo concolor* | 106 | 0 | 0 | 0 | 0 | 0 |
| *Hirundo rustica* | 124 | 1 | 0 | 0 | 1 | 1 |
| *Hirundo lucida* | 111 | 0 | 0 | 0 | 0 | 0 |
| *Hirundo angolensis* | 119 | 0 | 0 | 0 | 0 | 1 |
| *Hirundo tahitica* | 105 | 0 | 0 | 0 | 0 | 0 |
| *Hirundo neoxena* | 112 | 0 | 0 | 0 | 0 | 0 |
| *Hirundo albigularis* | 128 | 1 | 0 | 0 | 0 | 1 |
| *Hirundo aethiopica* | 106 | 0 | 0 | 0 | 0 | 0 |
| *Hirundo smithii* | 110 | 1 | 0 | 1 | 0 | 1 |
| *Hirundo nigrita* | 106 | 0 | 0 | 0 | 0 | 0 |
| *Hirundo leucosoma* | 99 | 0 | 0 | 0 | 0 | 0 |
| *Hirundo megaensis* | 102 | 0 | 1 | 1 | 0 | 1 |
| *Hirundo dimidiata* | 102 | 1 | 0 | 0 | 0 | 1 |
| *Hirundo atrocaerulea* | 113 | 1 | 1 | 1 | 1 | 1 |
| *Hirundo nigrorufa* | 112 | 1 | 0 | 0 | 1 | 0 |
| *Hirundo cucullata* | 125 | 1 | 0 | 0 | 0 | 1 |
| *Hirundo abyssinica* | 106 | 1 | 0 | 0 | 0 | 1 |
| *Hirundo semirufa* | 132 | 1 | 0 | 0 | 0 | 1 |
| *Hirundo senegalensis* | 144 | 0 | 0 | 0 | 0 | 1 |
| *Hirundo daurica* | 124 | 1 | 0 | 0 | 1 | 1 |
| *Hirundo striolata* | 124 | 0 | 0 | 0 | 0 | 0 |
| *Hirundo preussi* | 95 | 0 | 0 | 0 | 0 | 0 |
| *Hirundo rufigula* | 97 | 1 | 0 | 0 | 0 | 0 |
| *Hirundo spilodera* | 111 | 1 | 0 | 0 | 0 | 0 |
| *Hirundo fuliginosa* | 88 | 0 | 0 | 0 | 0 | 0 |
| *Petrochelidon pyrrhonota* | 109 | 1 | 0 | 0 | 0 | 0 |
| *Haplochelidon andecola* | 115 | 0 | 0 | 0 | 0 | 0 |
| *Petrochelidon fulva* | 102 | 1 | 0 | 0 | 0 | 0 |
| *Hirundo fluvicola* | 91 | 1 | 0 | 0 | 0 | 0 |
| *Hirundo ariel* | 91 | 1 | 0 | 0 | 0 | 0 |
| *Hirundo nigricans* | 107 | 1 | 0 | 0 | 0 | 0 |
| *Delichon urbicum* | 110 | 1 | 0 | 0 | 0 | 0 |
| *Delichon dasypus* | 108 | 1 | 0 | 0 | 0 | 0 |
| *Delichon nipalense* | 95 | 0 | 0 | 0 | 0 | 0 |

Figure S1 Examples of estimation of correlation coefficients between overall sexual dimorphism and extinction risk (a) and between sexual tail dimorphism and extinction risk (b) when applying a threshold model using all 72 species of hirundines (see methods section for detailed information). Mean correlation coefficients, indicated by broken lines, were 0.58 and 0.48, respectively (P_MCMC_ = 0.002 and 0.027, respectively).
